# Japan’s contribution to making global health architecture a top political agenda by leveraging the G7 presidency

**DOI:** 10.7189/jogh.08.020313

**Published:** 2018-12

**Authors:** Haruka Sakamoto, Satoshi Ezoe, Kotono Hara, Yui Sekitani, Keishi Abe, Haruhiko Inada, Takuma Kato, Kenichi Komada, Masami Miyakawa, Eiji Hinoshita, Hiroyuki Yamaya, Naoko Yamamoto, Sarah Krull Abe, Kenji Shibuya

**Affiliations:** 1 Department of Global Health Policy, University of Tokyo, Tokyo, Japan; 2 Ministry of Health, Labour and Welfare of Japan, Tokyo, Japan; 3 Ministry of Foreign Affairs of Japan, Tokyo, Japan; 4 National Institute of Infectious Disease, Japan, Tokyo, Japan

**Figure:**
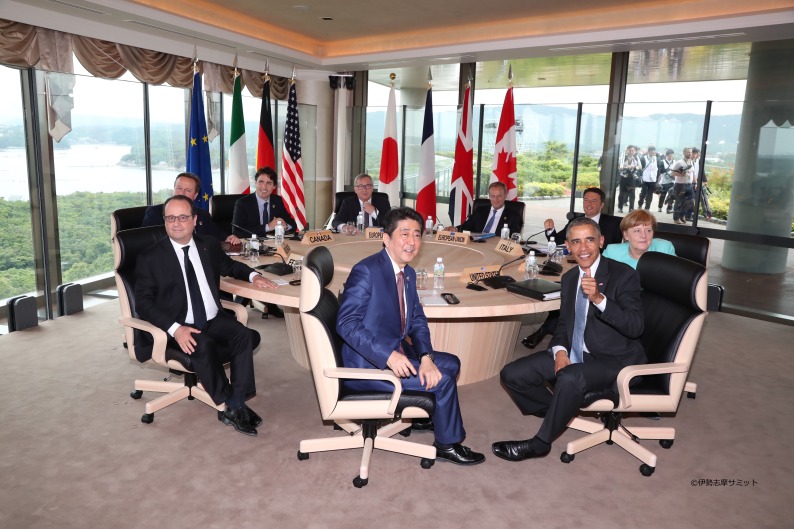
Photo: at the 42^nd^ G7 summit, Ise-Shima (from the Ministry of Foreign Affairs and Ministry of Health, Labour and Welfare; used with permission)

## CHANGING DYNAMICS IN GLOBAL HEALTH AND FUNDAMENTAL FRAGILITY OF GLOBAL HEALTH GOVERNANCE

Global health is currently at a crossroads. The majority of low- and middle- income countries are now suffering from a double burden of diseases. Compared with the Millennium Development Goals, the Sustainable Development Goals give less attention to health challenges. Additionally, there is also an increasing number of global issues competing for attention among policy makers, including downside risks to the global economy, terrorism, migration/refugees, and climate change. Consequently, the amount of Official Development Assistance for global health has stagnated in recent years [1]. These challenges are further confounded by newly emerging political and economic actors in global health arena.

Global health architecture (GHA) is defined as “the relationship between the many different actors engaged in global health and the processes through which they work together” by Kickbusch et al. [2]. The debates on GHA have been fueled by the complex interactions between health transitions, global health priorities, and uncertainties in global governance and economic prospects [2]. In particular, the Ebola outbreak in 2014 provided a wake-up call that drew the world’s attention to GHA. The World Health Organization (WHO), as the only United Nations (UN) agency specializing in health, was criticized for not handling the Ebola outbreak effectively and efficiently, which has evoked a series of debates and controversies on GHA [3]. In 1994, Jamison and colleagues proposed that the core functions of international global health organizations be the promotion of global public goods and the implementation of interventions to deal with international externalities [4]. Though global community including WHO has been making their efforts on GHA such as revision of International Health Regulations in 2007, the Ebola outbreak revealed the fundamental fragility of the existing governance, including that of the WHO, which could not handle these core functions: containment of viral transmission, vaccine provision, and the provision of other public goods [3].

In the midst of this transformation in global health, Japan hosted the G7 Ise-Shima summit in May 2016 and successfully set GHA as one of its priorities.

## HOW TO INCREASE POLICY COMMUNITY COHESION AMONG STAKEHOLDERS?

The key factor of Japan to successfully raise political awareness on GHA was that there was strong policy cohesion among stakeholders. There were four different actors: Japanese domestic stakeholders, G7 member states, non-G7 members and actors other than health sectors. First about actors in Japan, there are four major actors: the Cabinet Secretariat, Ministry of Foreign Affairs (MOFA), Ministry of Health, Labour and Welfare (MHLW), and Ministry of Finance (MOF). These ministries have slightly different views on and interests in GHA. Since health emergencies directly affect the health status of the Japanese citizenry, the MHLW expressed a strong interest in GHA at an early stage. MOFA emphasized the relevance of human security, which is defined by UN as “protecting the vital core of all human lives in ways that enhance human freedom and fulfilment,” and has been Japan’s core foreign policy. MOF focused on promoting the World Bank (WB) Group’s funding scheme initiatives (ie, Pandemic Emergency Facility (PEF) and International Development Associations) to respond to and prepare for health security. Since health security is strongly related to national, global, and human security, under Prime Minister Abe’s leadership, the Cabinet Secretariat and these three ministries successfully aligned around the goal of reinforcing GHA [5]. The three ministries and the Cabinet Secretariat constantly held joint meetings, with director-general level participants from each ministry, in order to share information and discuss how to consolidate Japan’s commitment in a unified manner.

Aside from Prime Minister Abe, Mr. Yasuhisa Shiozaki, then Minister for Health, Labour and Welfare, is a leading figure who has expressed enthusiasm about Japan’s leadership and contribution to global health. Under his leadership, the MHLW made a significant contribution to leading and promoting policy cohesion within the government. He established the Advisory Panel on Global Health in August 2015 so as to institutionalize a mechanism to develop global health policies within the MHLW. The Panel consisted of two working groups: human resources for global health policy-making and global health governance, which sought to make recommendations to the Japanese government [6]. This process contributed to the basis for discussions among Japanese stakeholders in reaching consensus on the global health agenda at the G7 Ise-Shima Summit.

Strong political support also came from Professor Keizo Takemi, member of the House of Councilors and a chairman on the Liberal Democratic Party’s Special Mission Committee for Global Health Strategy. As a champion for global health with a solid academic and policy-making background, Prof. Takemi has published internationally recognized papers that significantly influenced the previous G8 preparatory processes while also serving as the main advocate for global health issues through the track 2 process at previous G8 summits hosted in Japan. In 2016, he led the track 2 process for the G7 Ise-Shima Summit with a set of policy proposals from his working group [7]. Prof. Takemi also chairs round table meetings with the government and relevant private and civil society institutions, which serve to promote a mutual understanding of key global health issues, including those relevant to the G7.

As to the cohesion among G7 member states, GHA for future public health emergencies started to be shed light on at the 2015 G7 Elmau Summit in Germany. In the aftermath of the Ebola outbreaks, the WHO’s emergency reform plan was still at an early stage and therefore, there was virtually no strong opposition to include GHA for future pandemics into the G7 agenda; in fact, it was expected by the G7 members heads of state. Particularly the United States of America and Germany urged health security to be included as a G7 agenda item. The US has been promoting the Global Health Security Agenda (GHSA) and Germany highlighted the importance of health security at the Berlin Health Minister’s Meeting in 2015.

In order to elaborate and move forward the health-related agenda at the G7 Ise-Shima Summit in May 2016 and propose concrete actions to attain the goals described at the G7 Ise-Shima Leaders’ Declaration, the G7 Kobe Health Ministers’ Meeting was held in September, 2016, where four Asian Ministers as well as the WHO, UN Office for the Coordination of Humanitarian Affairs (UNOCHA), the WB and the Organisation for Economic Co-operation and Development (OECD) also joined discussions. Together with three official preparatory meetings, the meeting also contributed to increasing policy cohesion among G7 members both at head of state and health minister level.

In order to secure and deepen cohesion, it was important to have communication as extensively and effectively as possible, especially with non-G7 countries. Japan prepared several dialogue opportunities with these countries throughout its G7 presidency in 2016 including several side events at the 69th World Health Assembly (WHA), which resulting in enhanced mutual understanding of how the global community should rebuild and revamp GHA.

The WHA was an opportunity for Japan to disseminate G7 efforts towards GHA and reach out to health ministers and policy makers around the world, whereas the Tokyo International Conference on African Development (TICAD) in August 2016 was a platform to discuss GHA specifically with African leaders.

As the chair of the meeting’s thematic session for health, then Health Minister Shiozaki led an intense debate with the African heads of state and ministers, as well as leaders from international organizations. During the preparatory process, the MHLW had an extensive debate with the WB, the co-chair of the thematic session, regarding how to raise awareness for reinforcing GHA among African leaders, international organizations, and civil society organizations. Throughout this consultation process, they reached consensus on what should be done to prepare for and respond to future health crises, deepened the Nairobi Declaration and its implementation measures.

Lastly about actors other than health sectors, noteworthy influence came from foreign ministers. Public health emergencies were also highlighted as security issues for foreign ministers for the first time in the G7 Foreign Ministers’ Meeting Joint Communique (adopted at the G7 Hiroshima Foreign Ministers’ Meeting in 2016), which clearly mentioned the importance of collective efforts toward GHA.

## POLITICAL SURROUNDINGS AND FINANCIAL SITUATION ON GHA

The policy window and good global governance structure are key for attaining political attention and generally, a policy window is likely to open after major events such as disasters, discoveries, or forums [8]; the Ebola outbreak is no exception. Because it caused tremendous damage, amounting to a total of 28 616 cases and 11 301 deaths with global pandemic potential [9], it naturally attracted political attention, such as at the UN High-Level Meeting on the Response to the Ebola Virus Disease Outbreak in 2014 or in the creation of the UN Mission for Ebola Emergency Response (UNMEER). Under the UN Secretary-General (UNSG), the UN High-Level Panel on Global Response to Health Crises worked at the strongest power for opening the policy window by publishing an influential report, *Protecting Humanity From Future Health Crises*. Following the recommendations made by the Panel, the Global Health Crises Task Force was launched. Dr Shigeru Omi, the former WHO Regional Director for the Western Pacific Region participated in this task force with financial contributions from the Japanese government, aiming to enhance coordination between the work done by the task force and the preparatory process of the G7 Summit.

As to financial situation, at the time of the Ebola outbreak, the global community had neither adequate funding for outbreaks nor mechanisms of effectively disbursing financial resources [3]. However, some progress has been made, and the Japanese government has been the driving force of these improvements. The WHO’s Contingency Fund for Emergencies (CFE) and the WB’s Pandemic Financing Facility (PEF) were launched. CFE fills a critical gap from the onset of an emergency, which enables WHO to deploy experts and begin operations immediately. On the occasion of the G7 Ise-Shima Summit, Japanese Prime Minister Abe pledged a total of US$ 1.1 billion to global health institutes, including US$ 50 million to the WHO. At the G7 Finance Ministers and Central Bank Governors’ Meeting in Japan in 2016 where PEF was officially launched, the Government of Japan announced their financial commitment of US$ 50 million to this new facility.

Moreover, the Coalition for Epidemic Preparedness Innovations (CEPI) was also officially launched at the 2017 World Economic Forum, an international collective effort to create vaccines to combat future pandemics. Japan is a founding member of this new initiative, and has committed to contributing US$ 25 million per year in order to fund its programs.

## FUTURE DIRECTIONS ON GHA

Taking advantage of the G7 presidency in 2016, Japan has contributed to strengthening GHA for future public health crises through the involvement of notable Japanese political leaders and by enhancing community cohesion within and outside of G7 members.

Three leaders, Prime Minister Abe, which were echoed by then Health Minister Yasuhisa Shiozaki and Prof. Keizo Takemi, all contributed to strengthening collective efforts toward reinforcing GHA. The fact that powerful political leaders fully endorsed this agenda, echoed by the G7 leadership as well as the heads of WHO and the World Bank Group, remains an exceptional achievement in Japan’s history of global health policy making. As seen with the case of James Grant, former director of the UN Children’s Fund (UNICEF) who successfully drew global attention to children’s health [10], the emergence of strong political leadership helped generate a high level of political attention.

With regard to the political context, the severity and externality of the Ebola outbreak itself caused increased political attention, such as at the UN High-Level Meeting on the Response to the Ebola Virus Disease Outbreak and in several influential reports from WHO and academic institutions. As also seen with HIV/AIDS and NCDs, UN high-level meetings largely promoted the health agenda [11][12]. GHA was discussed at the UN high-level meeting, which in turn boosted GHA to the top of the global health agenda. Additionally, as seen in previous G7/G8 leaders meetings advancement of the global health agenda, Japan was also leading the political process and contributed to opening the political window: the G7 leaders at G7 Ise-Shima Summit, with health ministers at the 69th WHA, with leaders from African countries and international organizations at TICAD VI, and with G7 health ministers, WHO, and UNOCHA at the G7 Kobe Health Ministers’ Meeting.

Through G7 in 2016 and after, new financing schemes for CFE, PEF and CEPI was launched and these new mechanisms should be closely monitored and evaluated. In particular, effective and efficient use of financial resources is needed as scarce financial resources and tendency of waning political attention may hinder sustainability.

## References

[1] Institute for Health Metrics and Evaluation. Financing Global Health 2014: Shifts in funding as the MDG era closes. 2015. Available: http://www.healthdata.org/sites/default/files/files/policy_report/2015/FGH2014/IHME_PolicyReport_FGH_2014_1.pdf. Accessed: 26 March 2017.

[2] Kickbusch I, Lister G, Told M, Drager N. Global health diplomacy: Concepts, issues, actors, instruments, fora and cases. New York: Springer; 2012.

[3] Moon S, Sridhar D, Pate MA, Jha AK, Clinton C, Delaunay S (2015). Will Ebola change the game? Ten essential reforms before the next pandemic. the report of the Harvard-LSHTM Independent Panel on the Global Response to Ebola.. Lancet.

[4] Jamison DT, Frenk J, Knaul F (1998). International collective action in health: Objectives, functions, and rationale.. Lancet.

[5] Abe S (2015). Japan’s vision for a peaceful and healthier world.. Lancet.

[6] Japan Global Health Working Group (2016). Protecting human security: Proposals for the G7 Ise-Shima Summit in Japan.. Lancet.

[7] Takemi K (2016). Japan’s Global Health Strategy: Connecting development and security.. Asia-Pacific Rev.

[8] Shiffman J, Smith S (2007). Generation of political priority for global health initiatives: a framework and case study of maternal mortality.. Lancet.

[9] World Health Organization. WHO Ebola outbreak 2014-2015. 2016. Available: http://www.who.int/csr/disease/ebola/en/. Accessed: 26 March 2017.

[10] European Center for Peace and Development. Remembering Jim Grant. 2016. Available: http://www.ecpd.org.rs/pdf/2015/books/2016/2016_jim_grant.pdf. Accessed: 26 Marc 2017.

[11] Mamka Anyona R, De Courten M (2014). An analysis of the policy environment surrounding noncommunicable diseases risk factor surveillance in Kenya.. AIMS Public Health.

[12] Alfvén T, Erkkola T, Ghys PD, Padayachy J, Warner-Smith M, Rugg D (2017). Global AIDS Reporting-2001 to 2015: Lessons for monitoring the sustainable development goals.. AIDS Behav.

